# “TOGETHER WE ARE UNBEATABLE”: young sisters’ narration of a sibling’s cancer in personal blogs on the internet

**DOI:** 10.1080/17482631.2019.1586625

**Published:** 2019-03-27

**Authors:** Anneli Silvén Hagström, Teolinda Toft

**Affiliations:** a Faculty of Health and Occupational Studies/Department of' Social Work and Psychology, University of Gävle, Gävle, Sweden; b Clinical Psychology in Healthcare, Department of Children's and Women's Health, Uppsala University, Uppsala, Sweden

**Keywords:** Blog, coping, grief, identity, internet, sibling’s cancer, sisters narration, stigmatization, trauma, youth

## Abstract

**Purpose**: Siblings of children and young people diagnosed with cancer are commonly reluctant to talk about their experiences due to the circumstances of the illness situation. This article aims to bring voice to experience and inform practice by investigating what and how three young sisters narrate about their illness experiences in personal blogs on the Internet.

**Methods**: A narrative methodology for the analysis of life storytelling was applied primarily to investigate the sister’s coping strategies and support needs.

**Results**: The results show how the sisters constructed their own space for narration, with the main aims of expressing their feelings about the illness and seeking social support. The telling of their experiences along with encouraging comments from a supportive audience enabled a change in position from feeling neglected and silenced to being a recognized agent and caring sister. In addition, through their narrative coping the sisters went from powerless to powerful in their position in relation to cancer.

**Conclusion**: The results highlight the need for siblings to be able to narrate experience in a supportive context, where the processing of their relationship with the ill sister/brother should be understood as an important element of their coping with cancer and death.

## Introduction

Siblings of children and young people diagnosed with cancer (referred to below as *siblings*) commonly experience being positioned in a peripheral role within the family, where the main attention is on the ill sister/brother (Long, Marsland, Wright, & Hinds, 2015). They need to adjust to the ill sister or brother’s changing symptoms and the effects of the treatment, with consequences for their own life situation and wellbeing (Long & Marsland, 2011). Previous studies have reported that siblings experience loneliness and other strong emotions such as fear, worry, and sadness in relation to their sister/brother’s cancer (D’Urso, Mastroyannopoulou, & Kirby, 2016; Long et al., 2015). In addition, hospitalization means that siblings recurrently experience separation from and longing for the ill sister/brother and their parents (Yang, Mu, Sheng, Chen, & Hung, 2016). It also means that older siblings have to take on greater responsibilities in their home and everyday lives, as roles and routines in the family change and parents are less physically and emotionally available (Gerhardt, Lehmann, Long, & Alderfer, 2015; Tasker & Stonebridge, 2016). One study finds that the cancer experience affects all parts of a sibling’s life (Prchal & Landolt, 2012).

Siblings also report difficulties in talking about their experiences within their daily networks. They do not want people to pity them (Tasker & Stonebridge, 2016), or to be stigmatized through their identities being connected with cancer (Long et al., 2015). They find questions from individuals in their social networks uncomfortable, since they usually focus on the ill sister or brother and their parents, rather than the siblings’ own experiences and needs (Prchal & Landolt, 2012).

In response to what is understood as a highly demanding situation combined with inadequate support, siblings have been defined as a risk group for the development of short- and long-term psychosocial problems. They are claimed to be at increased risk of anxiety, depression, poorer quality of life, social problems, difficulties in academic achievement and post-traumatic symptoms (Gerhardt et al., 2015). This was particularly evident in one study of 78 siblings between 10 and 20 years old, where 32% reported moderate-to-severe symptoms of post-traumatic stress symptoms consistent with DSM-IV PTSD criteria. Such symptoms were *re-experiencing* the trauma through intrusive and distressing recollections of the event, *avoidance* of places, people, and activities that are reminders of the trauma, and *increased arousal*, meaning difficulty sleeping and concentrating and being easily angered (Alderfer, Labay, & Kazak, 2003). However, there are variations in experience and health outcomes among siblings and some report positive outcomes in terms of post-traumatic growth, such as increased maturity, empathy and resilience as well as closer relationships (Alderfer et al., 2009; D’Urso et al., 2016; Gerhardt et al., 2015).

The importance of providing psychosocial support to siblings has been established in several studies, together with the need for siblings to express their feelings and thoughts on their cancer experience (Gerhardt et al., 2015; Yang et al., 2016). However, reaching siblings with professional support is not always easy. For the reasons outlined above they may be reluctant to talk, while awareness of the severity of the disease might lead them to avoid putting additional strain on an already stressful situation for their ill sister or brother and their parents (D’Urso et al., 2016; Long et al., 2015; Yang et al., 2016). By contrast, and perhaps as an alternative to professional support, siblings in several studies have reported an urgent need to meet others with similar experiences (D’Urso et al., 2016; Tasker & Stonebridge, 2016).

Taken together, despite increased knowledge of the situation of siblings of children and young people diagnosed with cancer, we do not know enough about how these siblings manage their situation or the specific support they are looking for. This is crucial knowledge to enable the development of clinical interventions.

The Internet is a relatively new and popular social arena for communication and exchanges of support on demanding and sensitive life experiences, such as a sister or brother’s cancer. In Sweden, like many other Western countries, almost all young people have daily access to the Internet (Findahl & Davidsson, 2015; Lenhart, 2015). The Internet offers opportunities to communicate around the clock, and to remain anonymous while revealing personal details about one’s life, thereby eliminating the limitations and requirements associated with face-to-face situations. This communication has the potential to reach out to a broad audience of readers—and also to researchers. Hence, such self-initiated communication without the involvement and influence of the researcher represents naturally produced material, which offers new ways to investigate young people’s illness experiences (Eastham, 2011). Such first-person accounts and narratives about traumatic life events thus capture what is important to the teller rather than the researcher, and could be indicative for improving health care (Gibson et al., 2016).

## Aim

This study is grounded in a narrative theoretical perspective (Riessman, 2008), according to which the siblings of children and young people diagnosed with cancer are seen primarily as storytellers and social actors. Far from being passive victims, they are recognized as competent agents and narrators who use different and alternating strategies to manage their life situation, and possess the resources to tell others about their situation and seek support.

The aim of this article is to investigate *what* and *how* three young sisters narrate about their experiences of living with a sister or brother diagnosed with cancer in their personal blogs on the Internet. Of particular interest to improving family care and support practices that encounter young cancer patients and their families is how the sisters cope with the illness situation and what their support needs are.

## Personal blogs and the internet context

A weblog, or blog, is a frequently updated personal homepage, where an author posts entries that consist, for example, of texts, photographs, videos, and links. Readers of the blog can often comment on these entries. Unlike diary writing, the writing and reading of a blog happen simultaneously, and the audience response is an important factor in the blogging process (Karlsson, 2006). In this way, blogging can be understood as an activity that tries to synchronize experiences with—and evaluate them against—other people’s experiences (Van Dijck, 2004). The concepts of online and offline contexts are used to describe communication and social interaction via blogs. These contexts are closely connected and can interact (Stern, 2008). For example, if an entry on a blog receives positive feedback from the audience in an *online* context, this might lead to a change in the behaviour of the blog author in an *offline* context. The reverse is also possible.

An online context makes it easier for young people to discuss private concerns, perhaps most notably because of the anonymity and physical distance. It can also lead non-anonymous persons to feel relatively anonymous, and encourage people to tell more about themselves than they would in a traditional social setting (McKenna & Bargh, 2000). In addition, when interacting in an online context it is possible to take time, contemplate and rephrase without the built-in sensitivity of silence in face-to-face conversations.

## Method

### A narrative research approach

A narrative theoretical framework has guided this study, which emphasizes the connection between storytelling, meaning-making of personal experience and self-formation (Riessman, 2008). It argues that human beings are constantly engaged in storytelling to organize experience and identity in the light of reported events. Hence, people *interpret* events in their lives through narration, and *perform* who they are or claim to be from their learned experience and a constructed view of the world. This further implies that there is no such thing as a ready-made essential identity. Instead, there are “multiple selfhoods” consisting of a range of potential identity positions linked to different relationships, demands, obligations, and responsibilities (Mishler, 2009). Identity is thus a question of the positioning of *Self* and others, which is undertaken through storytelling.

Importantly, however, the stories we construct of personal experience are not constructed solely in the mind of the narrator. Instead, they are co-constructed with the listeners/respondents (ibid.), connected to a larger sociocultural context, and produced in line with or as resistance to the dominant narratives of that context (Andrews, 2004). Accordingly, all stories and retellings must be interpreted according to their own premises depending on how the narrator wishes to be understood by her/himself and others in the particular contexts of narration (Bamberg, 1997).

### Material

Material collection was undertaken with the intention of collecting siblings’ narratives about a sister or brother diagnosed with cancer in personal blogs on the Internet. The following inclusion criteria were used:

1. The blogs had to be written in Swedish.
2. The blogs had to be public with no password requirement.
3. The blogs had to be written by a sibling (15–25 years of age) of a young person (under 25 years of age) diagnosed with cancer.
4. The blog should address an ongoing process rather than reconstruct memories of illness.
5. The blogs had to contain rich narrative material to enable a narrative analysis.

The blogs were identified through a search engine for Swedish blogs, “Bloggportalen”. In total, 15 blogs written by siblings were identified, six of which had been written after the more intense phase of the illness or after the death of the sister/brother. In three of the blogs the author and/or the ill sibling were over the age of 25; and in three of the blogs there were insufficient entries to conduct a narrative analysis. In the end, three blogs were selected for the study, all of which were written by young women. From a gender perspective on coping, this is perhaps not surprising since young women are more prone to seek social support than young men (Frydenberg & Lewis, 1993). Consequently, this is a study that analyses three sisters’ narratives about a sibling’s cancer. The selected blogs are introduced briefly below.

*Emma’s blog*: Emma was 16 years old when she wrote the blog. Her younger brother, Fredrik (11 years old), had been diagnosed with a brain tumour. Emma began to write about Fredrik’s cancer just before his treatment was terminated. Her blog was active during Fredrik’s last four months of life and for seven months thereafter, and consisted of 69 posts.

*Lisa’s blog*: Lisa was 17 years old when she started to write the blog. Her younger sister, Maja (6 years old), was diagnosed with Lymphoma. Lisa began to write about Maja’s cancer nine months after her diagnosis, in the final phase of intense treatment. Her blog was active for almost three years and consisted of 198 posts.

*Anna’s blog*: Anna was 20 years old when she became a blog author. Her sister, Sara (21 years old), was diagnosed with a bone tumour. Sara initiated the blog. As she became increasingly affected by the cancer, Anna continued the blog by writing about Sara’s situation during treatment and palliative care. Anna wrote for two years and two months, 18 months of which was after Sara’s death. The analysis includes the 79 posts written by Anna.

### Analysis

A narrative analysis was conducted to investigate *what* the sisters narrated of their illness experiences in their blogged life stories and *how* they narrated it, with a particular focus on their coping strategies and support needs. The analysis was undertaken in the four steps described below primarily to capture the sisters’ overall life stories; their central storylines, the themes that their narratives revolved around, and the subject positions that the sisters took and attributed to others through their writing.

1. A case-centred analysis was conducted on each blog to capture the framing of the blogs and the sisters’ narrated life stories. Initially, this included the identification of the sisters’ articulated purpose for writing. The central storylines in their telling of their experience were then delineated (Mishler, 2009). This was achieved through a temporal linkage of separate narrative episodes into somewhat coherent storylines. These storylines were in turn narratively organized around a main plot or intrigue, depending on the points that the narrating sisters wanted to make (ibid.). Through the storylines it was also possible to identify so-called turning points—where life was described as having taken a new direction (Clausen, 1998). Such discontinuities, or changed life courses, described in connection with their sister/brother’s illness situation were particularly analyzed to identify the sisters’ associated meaning-making and self-formation.
2. A narrative thematic analysis (Riessman, 2008) was conducted of each blog to investigate *which* aspects of their illness experiences were specifically communicated, through the identification of central and reoccurring narrative themes in their stories.
3. Bamberg’s (1997) “performance-based pragmatic approach” was used to analyze *how* the sisters communicated their experiences to others and with what implications for their self-formation. This analysis was attentive to the sisters’ subject positioning on three different levels: (*a*) how the characters in the reported events were positioned in relation to one another; (*b*) how the narrator positioned herself in relation to the audience; and (*c*) how the narrator positioned herself with regard to the *Self* as part of identity construction.
4. Finally, the similarities and differences between the three cases were noted and analyzed.

The authors continually questioned and pragmatically validated their interpretations of the material in a dialogue (Riessman, 2008). In addition, the use of different but partly overlapping and complementary narrative approaches worked to validate the results—so-called validation by method triangulation (Silverman, 2005).

### Ethical considerations

The blogs are public material, since they are available for anyone to read and respond to on the Internet. At the same time, the blogs represent personal and sensitive material, which was produced without the blog authors’ awareness that it might be used for research (Markham & Buchanan, 2012). All the blogs had been completed at the time of analysis and lacked any reliable contact information, which made it difficult to obtain informed consent from the blog authors. Instead, measures in line with the ethical recommendations in netnography^1^ were taken to protect the integrity of the blog authors (ibid.). These include the omission of real names and alteration of any personal information that might reveal identities, such as ages, references to years and locations, and so on. In addition, the excerpts have been translated from Swedish to English, which excludes the possibility of searching for verbatim excerpts on the Internet. The study was approved by the Regional Ethical Review Board in Uppsala, Sweden (2014/251).

## Results

### The blog context as a “therapeutic room”

A common characteristic of the blogs is that they were sites consisting of diary entries and photographs, which together created a personal space for the narration of experience—or a “therapeutic room” (see Silvén Hagström, 2017)—to which readers were invited in order to share and respond to the sisters’ writings. The affective side of this communication was first and foremost mediated through the photographs. These portrayed the sisters’ close relationship with their ill siblings, as well as their struggle against cancer in up and down periods during treatment, and in two cases also during palliative care. The readers could therefore witness the progression of the siblings’ illnesses, for example, by meeting their painfully sad and worried eyes and watching extreme bodily changes such as hair loss, swollen faces due to cortisone treatment and severe weight loss during the terminal phase. Hence, the photographs helped create an emotional bond between the readers and the ill sibling, and promote empathy for him or her and the blogging sister’s situation. This emotional process was usually manifest through a strong commitment and encouraging comments in response.

### The framing of the blog narratives

The framings of the blog narratives were analyzed separately to identify the aim of each blogging sister in writing from her articulated position and need. Emma opened her blog with the story of how her brother Fredrik fell ill in 2006:

Hi, I created this blog to write about my little brother’s cancer and nothing else. On June 5th, 2006 my then 8-year old brother went to hospital with a headache […]. On my birthday, he received his diagnosis. He had a brain tumor. He was immediately hospitalized and medicated with high doses of cytostatic. Fredrik’s biggest worry was losing his hair. He went in and out of hospital and I started to live more and more alone at home.

In her writing, Emma positioned herself as lonely. She described this as a direct consequence of Fredrik’s cancer and she demonstrated her need to tell of her illness experience when she informed readers that Fredrik’s cancer would be the topic of her blog. A special desire was expressed: to reach out to other siblings of a sister or brother with cancer, in the hope that her blogging could be of support to them, as it probably would be to her too if a sibling with a similar experience responded:

I hope many siblings of ill children will read this. They’ll recognize themselves the most. So I want to ask all of you readers to forward this blog so that it eventually reaches them!

In a similar way, Lisa wrote in her blog introduction that she wanted to share her experience of the treatment of her 6-year old sister, Maja, for lymphoma:

I have today January 15th persuaded myself to create my own blog to share my thoughts and views on my life in the nine months that have passed since my little sister, Maja 6, fell ill with lymphoma. I’ll try to update as often as I can and have the time […]. I won’t force anyone to read it, but would appreciate it if someone did!

In her first entry in her and Sara’s shared blog, Anna also expressed a wish to get in touch with other siblings of a sister or brother with cancer to counteract her feelings of loneliness: “Hopefully also those who are or have been in the same situation as us can find their way here and gain support. It always feels comforting to know that you are not alone”. When Anna later took on the blog herself, after Sara’s death, she expressed a similar need to connect to grieving siblings due to a brother or sister’s cancer:

For a long time, I’ve been quite desperate and searched and searched for someone who is or has been in the same situation […]. Although you’d never wish for anyone else to be in the same situation, it’d be nice to find someone and feel that you’re not alone. Someone to share things with, who feels the same as I do; the same emptiness and the perception of being half a person without the other.

Together, the author sisters’ framing of their blog narratives demonstrated an acute need to tell of their experiences, with the primary purpose of relieving distress and processing the traumatic events of each sibling’s cancer and their current circumstances. However, a second and perhaps equally important purpose was their evident need to seek recognition from other siblings of a sister or brother suffering from cancer, or who had died as consequence. The latter was explained as important to reassure themselves that they were not alone, and so they could contribute to providing support.

### From normality to a life of chaos and struggle

In all three sisters’ life stories, their sibling’s diagnosis of cancer was described as a turning point. They all portrayed how they immediately had to adjust to a chaotic family situation and an increasingly demanding daily life. Emma’s entry is a case in point:

Shit, you forget what it’s like to come home after school: all the family out on their own for the whole day. You sit down and eat and talk about what the day has been like, and then you’re off to table tennis practice. Now it’s a life of medication, lack of money, food at odd times, fast food in the microwave, anxiety, grief and choking back tears. When you look at it from the outside it sounds so sick.

In her narrative, Emma contrasted her current situation with the ordinary life that she used to know. She portrayed a shattered family life, fully adjusted to Fredrik’s illness and treatment, with a lack of daily routine and meeting opportunities, and a strained economic situation, as well as her own lonely position in coping with it all. At the same time, she let readers understand that in normal circumstances this is a family in which people care about each other; they take time together to contemplate what the day has been like. In her description of ordinary life, moreover, Emma positioned herself as a normal teenager who had the motivation and time to develop interests and relationships outside of the home.

Lisa, in a similar way, reflected on how her younger sister Maja’s cancer dramatically affected her family:

The things I’ve been through have changed big parts of my life. I have a family, a mum and dad, two brothers and two sisters. We’ve always got along well, didn’t fight that much. We took care of each other. We were a normal family, with work and school. To be honest, we were quite a calm family. But this changed fast. Our calm family changed into a family struggling to survive a war […]. Suddenly it was as if the Second World War came into our lives, and the ground disappeared from under our feet.

Here too the family was described as a normally supportive and caring environment, which changed when all focus and energy were concentrated on the struggle with Maja’s cancer. Just like Emma, Lisa described how she first and foremost coped by *not* talking about Maja’s cancer. In a reflection on why she would easily cry and lose her temper she articulated: “Probably it is because I must digest my feelings, swallow and shut up. Or because I don’t want to burden others around me with my thoughts. Come on, Lisa. Damnit!”.

Unlike the others, Anna did not mention how her family life had changed due to her sister Sara’s cancer. Instead, she described how she herself was struggling to comprehend the new situation, cope emotionally and go on living. In common with the others, however, she described how she primarily coped by keeping her feelings and thoughts to herself while trying to act her “normal” *Self*:

In this terrible journey that our family has been through, you’ve been struggling with your emotions daily. You don’t recognize your own body and yourself at all, among other things. […] You talk to others, you do things to stop yourself from thinking, you do what’s normal, but I feel that I’m not there. I can speak without hearing what I say, laugh without knowing why I’m laughing, walk without knowing where I’m going. […] As I said, it isall so horribly unreal, but at the same time totally real, but… I still don’t grasp it. As if on the outside the body forces itself to look normal, when the inside is broken and crushed.

All the sisters described a position of being deeply affected by illness-related circumstances outside of their immediate control—both psychologically and in their daily life routines—and how they expected themselves, and perhaps were also expected by others, to adjust accordingly. They described how they had lived a normal life and how life in the wake of cancer took a divergent and unknown direction characterized by pain and uncertainty. This phenomenon—how the illness as a “disruptive event” divides life into a *before* and *after—*has been called a “biographical disruption” (Bury, 1982). In his study of chronically ill patients’ autobiographies, Bury identified disruptions and responses on three different levels: (*a*) disruptions of previously taken-for-granted assumptions and behaviours; (*b*) disruptions of explanatory systems, which require a fundamental rethink of the person’s biography and self-perception; and (*c*) responses to disruption, involving the mobilization of social resources in facing an altered life situation (pp: 169–170). In the following, it becomes obvious that these aspects of a disrupted biography also apply to the narratives of these sisters who were indirectly affected by a sibling’s cancer. Hence, through their blogging the sisters sought to reconstruct meaning and identity, and their blogging should be seen as an act of mobilizing social support.

### The loss of meaning and identity

In this demanding situation, with few opportunities to talk and a reported need to do just that, the sisters’ psychological processing of their sibling’s cancer became the focus of their blogs. Emma, for example, questioned the meaning of Fredrik’s cancer: “As I said, I hate this more than anything else and it just doesn’t seem to have any meaning”. From a confused identity position, Lisa reflected on how she could no longer identify herself with the sociable and talkative person that she used to be before Maja’s cancer:

I’ve always been a sociable person who usually expressed my opinion. I must admit, I was quite chatty. Instead of this normal Lisa, I became Lisa who didn’t have her feet on the ground. I became very insecure and didn’t know what life had in store for me. I became introverted. My life became chaotic. At home I was the Lisa I dared to be. In school, I was another.

In her self-description, Lisa showed how she was emotionally affected by Maja’s cancer and was therefore no longer the outgoing and positive spirit that she used to be. At the same time, she pointed to external circumstances as she made clear that different identity positions were available depending on the context. At home she was who “she dared to be”, indicating that this was conditioned by Maja’s illness situation.

After the death of Sara, Anna also began to question her life meaning:

It’s claimed that things have a meaning. But what the hell is the point of this? To walk in the city; streets, shops, places where we used to walk together […]. Together, you and I. To visit someone and to be reminded of when I was here the last time you were alive. The last time I sat on this bench you were healthy. The last time I walked down those stairs life was normal.

Anna recurrently questioned why she was alive and not Sara. Her search for meaning also involved questions about her future life:

I’m wondering what it will be like later, in a while. How it will come to shape me and how I’ll carry this with me in life. How it will feel to carry this heavy load through life. It will always be with me. Perhaps I’ll become stronger and carry it better, although it will still weigh the same.

When it comes to grief, this loss of meaning in the mourner’s life and self-story has been conceptualized as a “crisis of meaning” (see Neimeyer & Sands, 2011), and mourners’ ensuing need to narrate experience in order to reconstruct and reaffirm fundamental beliefs and identities has been highlighted as essential for the processing of loss.

Thus, the lonely position that the sisters revealed in their blog introductions is also apparent in their narration of experience. In all cases, the blog became a social arena where the sisters’ previously silenced voices could be heard and recognized, and their experiences of their siblings’ cancers testified as real in relation to an empathic and active audience (Fivush, 2010). The blogs thus facilitated a narrative processing of their trauma, so that new meanings and identities could be explored, negotiated, and reconstructed.

### The experience of stigmatization

Another aspect of the sisters’ lonely position in coping with their siblings’ cancer was a shared experience of stigmatization in their social networks. According to Goffman (1963/1990), stigma is present in all social interactions and involves a process whereby people socially read, interpret and negotiate the meaning of others’ behaviour, and categorize others—and the *Self*—in relation to “normality” and “deviancy”. In the blog narratives, the sisters reported negative responses from others, which we mainly understand as a product of their insecurity and need to distance themselves from a dreadful subject such as a child’s or young person’s life threatening disease, rather than of harmful intentions. The sisters’ social reading of others’ responses, however, may have contributed to experiences of differentiation and stigmatized self-formation (Goffman, 1963/1990). Emma, for example, questioned how individuals in her vicinity responded to her in her sibling position, and told of her subsequent anger and disappointment:

Honestly, I don’t understand people. When you meet familiar people in the grocery store, they turn their backs on you and ignore the problem. We’ve not asked you to listen to us, or our problems, or to send us gifts or whatever you imagine. We just want to feel accepted in our situation when it’s like this! Everyone is such a damned coward. To ignore problems, to go on believing that you live in a perfect world. At least that’s what you want to think.

Emma testified to how she was treated differently because of Fredrik’s cancer and how her social network, which could have provided support, added further stress to her situation.

Similarly, Lisa reported how her peers at school responded to the news of Maja’s cancer with uncertainty and distancing, which reinforced her lonely sibling position:

The rumor of what had happened to our family spread rapidly in school and suddenly people started treating me differently. As soon as I entered the hallway or classroom, everyone stopped and looked at me. Then they turned away without a word. Seriously, I felt like I wanted to vomit.

Lisa clarified to her readers that loneliness is the main feeling in the sibling position:

Nobody understood how I was doing or how I felt. It was impossible to put my feelings into words. All I can say is that I was lonely. I was full of loneliness and had never felt so invisible and small. It’s a common feeling when you’re the sibling of a child with cancer. It’s easy to forget about you and you become invisible. It’s just the way it is.

A change in this regard, however, came when Lisa was invited to a sibling camp organized by the local hospital. She described how she could find recognition and support for the first time:

I thought I was the loneliest person in the world, with a sister who was ill with cancer, but when I arrived at the camp I soon realized that I was not alone. I’m far from alone. I have never been alone and I will never be alone. There are others like me, or ‘others like me’ sounds a bit strange and wrong, but anyhow. I’m not the only one in this situation. I share feelings with quite a few young people in Sweden. Not very many, but they are a few at least. It’s good to keep in mind.

Her story shows that Lisa’s participation in this supportive context functioned to de-stigmatize the sibling experience of cancer, and that she could normalize herself. Indeed, just such an effect has been reported in evaluative studies of professionally led sibling support groups (see Nolbris, Abrahamsson, Hellström, Olofsson, & Enskär, 2010).

Anna also reflected on her strange experience of being invisible to others:

Of course, it feels strange and awkward when someone you always said hi to just walks by and sees you as a ghost, in that moment at least. Then you think to yourself, ‘well okay my life is not like it used to be before’ and reality dawns. I have probably done the same myself when friends have been in difficult situations, because you don’t know how to react. Then I can turn it around and want to be a ghost. Nobody sees me and I’m invisible. You wrestle with these things daily.

Although Anna is affected by the negative responses of her peers, she interprets them as a sign of insecurity with her changed situation. In the blog context, by contrast, the sisters received confirmation from readers that they were “normal” and “good”, or even “mature” and exceptionally “gifted”, coping well with the situation surrounding the illness.

### The “fight story” and the “love story” as coping strategies

In the material, a primary strategy for coping with cancer among the sisters was to construct a “fight story” together with the ill sibling. This storyline was constructed in line with the dominant modernist and medical perspective of a “restitution narrative” (Frank, 2013, p. 75). The main plot from a patient perspective is: ‘Yesterday I was healthy, today I’m sick, but tomorrow I’ll be healthy again’ (p. 77). In this story the medicine owns the voice and the intrigue, and is positioned as the hero who will cure the illness. The patient, by contrast, is positioned as a passive victim of circumstance who lacks voice. The patient’s close ones have no other position than that of passive bystanders. As resistance to this passivity and victimization, these sisters regained power by creating a counter-narrative (Andrews, 2004), where they owned the intrigue, and where their ill siblings were positioned as the heroes. These fight stories, however, show how the sisters and their ill siblings drew on battle metaphors. They correspond with another dominant discourse of “the fight” or “crusade” against cancer, in which cancer patients should shoulder the responsibility of “getting well” (Sontag, 1978, pp. 57–58). Hence, armed with all their power, they focused on defeating the evil monster of cancer.

This time we will start with Anna. Shortly after Sara was diagnosed with cancer, Anna exclaimed in capital letters: “TOGETHER WE ARE UNBEATABLE”. She continued: “…and we willmanage this. We are so strong and with all the positive energy, magical energy, this will be good. You’re the best in the world”. In a follow-up post she announced to Sara and their audience:

It hurts so bad inside to watch you go through this. I feel so helpless. My beloved sister, the most important in my life, the one who I share EVERYTHING with. Sara, I’ll fight with you and together we’re unbeatable, because I don’t know anyone who has the power that you and I have together. We’re one and we’ll fix this. We’ll NEVER give up!!!

This fight story was reproduced many times in Anna’s blog during Sara’s treatment. The main storyline was that they—as “team Sara”—would battle cancer. A love story was constructed that worked to strengthen their bonds with each other. When the news arrived that Sara’s cancer was untreatable, Anna displayed devastation and confusion that the meaning in the fight story was lost: “This was the worst that could happen. We hoped and fought and did all we could, without getting anything back. Why?” When medicine declares that all hope for recovery is gone, the restitution narrative fails, and usually no alternative narrative framings are provided by medicine that can cover the patients and their families’ experiences (Frank, 2013). Anna was thus left in a position where she could either become a “narrative wreck” (ibid., pp. 53–56), or fall back on their alternative “quest narrative” (p. 115)—the love story.

In the aftermath of Sara’s death, Anna nourished what can be understood as a “continued bond” (Klass, Silverman, & Nickman, 1996) with Sara. That is, in contrast to the dominant modernist belief that “normal” and “healthy” grief involves a process of “letting go” before it can be resolved, the post-modernist view in continuing bond theory accentuates how grievers can construct inner representations of their deceased loved ones through which they sustain their attachment. Accordingly, Anna strenuously reproduced her love story with Sara as it carried a powerful meaning for her experience, which probably helped in her reorientation of their relationship. Her meaning reconstruction in grief thus read: since our love is not conditioned by time and space and “love conquers all”—even death—we are undefeatable. Moreover, Anna continued a dialogue with Sara that they used to have when she was alive:

I wanted to do a heart for Sara, as a proof that she’s always with me in all nice/important/special/meaningful places. I like doing hearts for Sara. We always made hearts for each other. Every time I left the hospital to go home for a while or to sleep, I stood in the door and made a heart with my hands and she did one back. Every time for as long as she could use her hands. Then I continued and will do it forever. So I’m thinking that every time I see a nice place or do anything important and so on, I’ll still do a heart for her. That feels good.

In the same way, Lisa wrote about Maja’s struggle against cancer in the form of a fight story. She positioned Maja as a solitary warrior and described how Maja’s lovable and caring personality gave the whole family strength to support her. After Maja’s treatment had been successful, Lisa again underlined her extraordinary personality traits and suggested they were a major contributory factor in her survival. Here too, a love story ran parallel with the fight story:

But I don’t think she realizes what an angel she is. When she was given chemotherapy, she made the rest of the family happy through her energy and her lovely laughter, even though she was really suffering, felt sick and just wanted to rest. She’s always there for others and has so much to give of herself. She’s amazing. And I love her so much. […] I’m so damn proud of what Maja has shown us. She has certainly proved that she can survive anything, whatever comes along. That’s truly my sister.
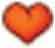

Emma began blogging at a turning point in her and Fredrik’s fight story, when they had just got the news that he would only be receiving palliative care from now on. Until then, Fredrik had been positioned as the brave and wise warrior David fighting an unequal struggle against Goliath. After the doctors terminated all active treatment, for a long period Emma expressed confusion and hope that perhaps Fredrik would defy the odds, as she saw him struggle and live longer than expected. Emma and Fredrik also co-constructed a love story. Emma portrayed a reassuring dialogue between the two during Fredrik’s last days of life:

A couple of nights ago he told me he could die for me, and that I was the best sister in the world. I told him I was the one who could die for him! He said I didn’t have enough experience, that my soul was too young. Then he said I should not be afraid to grow up, because he will watch over me.

When Fredrik died, Emma continued to articulate their love story, based on their shared meaning of a never-ending bond of love that would survive cancer. She announced that she would live to the fullest for them both. Through their shared meaning-making of death, Emma could thus incorporate the idea of Fredrik as a resource in her continued life:

I love you Fredrik, I’m so proud of you. You’re the best I know; you had been fighting for so long and the hope didn’t leave you. I’ll live for you; I’m going to make you proud. You’re so much wiser than I am. I couldn’t have had a better little brother. We’ll always be sister and brother. The same flesh and blood. FOREVER. And soon I’ll tattoo Fredrik on my left wrist (closest to the heart). So every time I make a decision, I’ll look at my wrist and think of what you would do.

In the two cases where the ill sibling died, the continued bonds with the deceased sister or brother served the need to remain attached with the deceased sibling, and to construct meaning from the loss experience. This communication about a continuing bond was articulated to the audience of readers, and also directly to the deceased sibling in a post-loss communication that was facilitated by the blog context (c.f. Silvén Hagström, 2018).

### The identity position of the “loving and caring sister”

A shared feature of the blog narratives was that they frequently reported on the health status of the ill sibling, with descriptions of daily life that positioned the blog authors as loving and caring sisters. Although the illness situation was described as extremely demanding, which might challenge the relationship with the ill sibling, a dominant theme was the sisters’ gratitude for being able to be close to their siblings, and to be a vital part of their care. As mentioned, Anna positioned herself as part of “team Sara”. She also described how she prayed and convinced herself that she could magically send energy to Sara to make her well:

Every night I lie awake and clasp my hands together and pray that Sara will be well. And yesterday I got a really strong feeling that this is going very well. Now I live on that feeling […]. Today I held her hand the whole time and thought to myself that I had a superpower that was being sent to her through her whole body, and that it will help her.

After Sara’s death, Anna thought back on the last days she spent with her on the palliative care ward. Anna’s only focus was to be a support to Sara, by sharing a last joyful moment based on her fascination with animated movies, and showing her endless love:

If we’d not seen Aladdin, Tarzan, Finding Nemo, Ariel, the Lion King and all the other wonderful movies a million times before, we certainly have now. She loved watching the movies again and again […]. The movies, the candy and the ice cream, it was the best. And we were so happy that she could be with us and that she could watch the movies and eat whatever she wanted. […] We told her every day thousands of times how much we loved her. And I told her all the time that I’ll let the whole world know how proud I am to be her sister. Every day.

Lisa similarly declared that her main need was to be a support to Maja: “You are so scared and totally confused. The world feels like it has been sunken underground. You don’t know how to manage the situation, but I do know that I willbe here to support my sister”.

In her daily reports, Emma also showed how she was torn between her needs to take an active part in Fredrik’s terminal care, to manage school and to do age-appropriate activities with her friends. However, she made clear to her readers that Fredrik’s needs were her main priority; something that recurrently ended with difficulties in achieving her academic goals, and sadness that she could not meet her own teenage needs. Supporting Fredrik was also described as a demanding task:

Fredrik’s mood is going up and down. Shit, lots of mood changes right now. Some days he wants you to leave: other days you’re not allowed. It’s understandable and I’m not annoyed at him. I’m lucky we have this relationship. I don’t think that many siblings have a relationship like ours.

Emma’s descriptions of too-demanding responsibilities at home should not be understood only in relation to her own unsatisfied wishes and needs, but also as a result of her vulnerable family situation. Her single mother struggled to take parental responsibility and was torn between her different tasks, and Fredrik could suffer as a consequence:

I’ve started to feel guilty when I stay away now. I try to think that he’s my little brother, I’m not his parent. I’m not supposed to have this responsibility that I have, but it doesn’t always work. And all I can think of is how Fredrik is lying there miserable screaming in the living room. The dogs worry and no one takes them out so that they don’t have to hear it. When I’m home I take care of Fredrik and cook fast as hell. When I’m not at home my mum needs to do it ALL. She must be in the kitchen cooking and hear him cry in pain, or she stays with him and listens to his cries of hunger. You wish that you had 10 arms or something.

Emma is the only sister who provides insights into the disadvantages of her sibling’s cancer in relation to her own needs, but even in her blog this constitutes an exception to her main positioning. The role of “the loving and caring sister” is the most common self-positioning. This illustrates the sisters’ need to be close to their ill sister or brother and take an active part in their care. However, this is also the most expected positioning from a sociocultural perspective, since the sisters are keen to present themselves as morally acceptable individuals. It would probably have been too difficult for the sisters, even in an accepting context like their own blog, to disclose all the aspects of their experience.

### When the ill sibling recovered or died

In all the blog narratives, the news of the ill sibling’s cancer was depicted as a turning point or biographical disruption in life while the narrative construction of experience sought to link together the *before-* and *after*-life. Similarly, the news that either the sibling’s treatment had succeeded or his or her condition was incurable represented another turning point. The intense struggle for recovery was finally rewarded, or another struggle had begun—to survive the severe loss of a beloved sibling.

Emma described how she was constantly hit by painful memories of Fredrik’s illness and death, which even made her change her daily routines:

I really hate to take the bus home after school because I always did that when Fredrik was alive. You sat on the first bus, changed bus and waited for 15 minutes. I arrived home 45 minutes later. Same thing every day. Every day I went down the hill from the station and I knew that inside the apartment Fredrik would be lying there with all his pain. As soon as you sit on the bus it feels like you rewind time. I don’t know what’s worse, to come home to an apartment filled with pain and worry, or to an apartment filled with emptiness. I’ve started to pay to go by train instead, to break the pattern.

In Emma’s bereavement narrative she turned directly to her audience of readers to instruct them on how they should *not* try to comfort her with the cliché that “time heals all wounds”:

Never say that it will be calmer in my life, that I’ll start to feel better soon! Because I climb and climb but there is always an obstacle which hinders me from going forward. And in the end, I fall down to zero again […]. Hate this fucking shit!

In so doing, she claimed the right to her chaotic position in grief, and opposed a position as victim who could be exposed to others’ well-meant assurances that things would eventually get back to normal (c.f. “chaos narrative” Frank, 2013, p. 97).

Anna, like Emma, struggled to make sense of Sara’s cancer and subsequent death:

Today it’s 3 months since Sara left earth, 3 months? It still feels so close, every second. I still feel that I walk around and hide my grief, because I can’t get what happened. And I’ve been thinking that I might never get it. The bubble you lived in for a year, you thought it would explode, but it’s somehow even thicker. And the body does not want to and cannot understand. I have so much inside. It feels so deep and heavy that I just want to keep it to myself. I just want to be shallow and talk about clothes, make-up and hair, or how well a girl on [Swedish] Idol sings.

Anna likened the period of Sara’s cancer to a “bubble” and described how after Sara’s death, she was repeatedly thrown back in time by intrusive and painful recollections:

You’ve lived in a bubble and it’s just now that you begin to realize how terribly ill Sara was. It’s the hardest thing, that everything comes back and that those eight months of our lives are being replayed in the head 100 times non-stop.

Anna’s descriptions of her struggle with intrusive reminders of Sara’s illness situation and death, just like Emma’s conscious strategies for avoiding previously routine-bound locations, are consistent with symptoms of post-traumatic stress (see Alderfer et al., 2003; Gerhardt et al., 2015). Through their writing, both Anna and Emma probably aimed to inform their readers about their response to loss, but also to validate their reactions in relation to others with similar experiences.

Lisa was the only sister to experience her sibling’s recovery from cancer. However, despite the news of Maja’s positive tests results, she described a gnawing worry that Maja would suffer a relapse during the follow-up period:

On Monday, it’s time to visit the hospital again. Maja is going to have a blood test. Is there any reason to worry? Should I be afraid? Of course I’m always scared and worried at times like this, of course I’m terrified of the results, of course I don’t dare to hope for the best.

Her concern was expressed repeatedly and intensified around Maja’s health check-ups. Instead of coping with Maja’s acute illness, Lisa now needed to cope with insecurity about Maja’s future health, as a new countdown had begun:

Don’t know why, but it’s like all the emotions surface afterwards. Before I thought it’s all good. Maja is well and everyone should be happy. But dad reminded me: Maja’s not healthy. Four and a half years to go before she can be declared healthy. That’s a long time. And if I walk around dreading what will happen in the coming years…I don’t want that. But it’s just to bite the bullet and swallow it all. I live in the moment and can’t do more than accept the situation. I’ll wait and wait for those words that will make my best day.

When the blogging ended each author found herself in a different situation. However, the blogging process evidently facilitated their coping and search for meaning and identity in the wake of the cancer experience. They all described how they would continue their “struggle” with grief or worry about relapses outside the blogging context, and that they no longer felt the need to narrate their experiences in public. Perhaps this indicates that they had reached a new plateau where their needs had changed, and had perhaps found coping strategies in the online context that could be used in their future offline lives.

### The responses of other sibling readers to the blog

The interaction between the author sisters and the readers was intense in all three blogs, and represented an important component of the blogging process. Most readers, however, had not been affected by cancer themselves. Instead, they took up a supportive stance and formed an empathic bond with the author sisters and their siblings. By contrast, other siblings in a similar situation responded with their own narrated experience. This sister’s response is a case in point. It shows how reading a blog can lead to recognition and counteract loneliness—in line with the author sisters’ initial assumptions and aims:

Hi Emma! I would like to give you a big hug. I know exactly what you’re going through. My little brother died when I was 18, having been ill for 4 years. Today I’m 34 and mother of two small girls. When I read your blog, I’m thrown back in time 16 years. I recognize the anger, the grief and the powerlessness, the school with the insurmountable demands and little empathy, and all the people who understand zero! To be alone, and everything you must tackle at home, anxious parents, etc. I don’t have to tell you, because you know it all! Hugs, Linda

The responding sister in retrospect recognizes herself in Emma’s writing and normalizes the lonely sibling position, with its inbuilt challenging demands. This sister reader similarly recognized herself, her family and their own process of treatment and hope, despair and death:

My sister became very ill and was hospitalized. She left us in June 2011, four weeks after the news of her cancer. She left us with a relieved smile on her face. 24 years old and in the middle of life. Sick to see that while we went on in our bubble during all this time, you a different family was struggling on and went through the exact same thing at another hospital at another location in the country. When we sat at the Christmas table and said: ‘Think of next Christmas, when you’re healthy and can eat and drink as much as you want’, you sat there too and probably had the same thought. That next Christmas it’ll all be over. Then we’ll all feel good, because there was nothing else. Of course we’ll overcome, together. As we’ve always done.

The chat blog communication can thus serve as a tool for getting in contact with siblings in similar situations and for normalizing the sibling position in relation to young people with cancer. When these kinds of collective experiences are linked together in a public blog context, a distribution of lived knowledge is also achieved, which can contribute to an increased understanding also among those not affected by cancer, thereby counteracting stigmatizing responses to cancer (c.f. Silvén Hagström, 2017).

## Discussion

This study analyzed three young sisters’ blog narratives about their experiences of a sibling’s cancer. All the narratives covered a period of acute illness. The material differed, however, in that one blog also covered the post-illness situation after a younger sister’s successful treatment, while the other two covered the acute grief after a younger brother’s or a similarly aged sister’s death. Nonetheless, a shared purpose and thematic were identified in the blog narratives. The blog writing was motivated from the position of what was described as a lonely and silenced sibling. All three sisters described how they had tried to cope with the illness mostly by keeping their feelings and thoughts to themselves, from a fear of being stigmatized and a reluctance to put extra pressure on parents. Through their blogging, the sisters resisted such positioning and constructed a personal space for narration, where it became possible to articulate experiences and to invite responses from a supportive audience. They wished to relieve distress and make sense of the cancer experience through the narration, but also to connect with other siblings in similar situations. It was assumed that the latter would counteract loneliness through recognition. The blog narration thus enabled a change in position: from one where the sisters felt neglected and silenced, to one of a recognized agent and caring sister for their ill sister or brother.

In addition, the sisters moved from a powerless to a powerful position in relation to cancer by using two main narrative coping strategies: “The fight story” and the “love story”. The fight story was during the illness phase. It aimed to gather the strength to battle cancer together with the ill sibling. When this narrative lost its power—in the cases where treatment was ended—the sisters instead co-constructed a love story with their ill sibling. This functioned as a counter-narrative to the general understanding that they had been defeated by cancer (Andrews, 2004). Hence, cancer could never “win” the struggle because love conquers all—even death. Both the sisters who lost a sibling to cancer continued to articulate this love story to their deceased sister/brother in an ongoing communication in the blog. This demonstrated their “continued bond” (Klass et al., 1996) post-loss, which seemed to facilitate their processing of grief.

The results correspond with previous research, which has demonstrated that siblings of young people diagnosed with cancer have too-demanding responsibilities imposed on their daily lives (e.g., Gerhardt et al., 2015; Yang et al., 2016). For example, the sisters disclosed how they spent time alone at home during hospitalizations, took over household chores, and participated in the direct care of the ill sibling. Ultimately, they showed how they needed to manage an overall stressful life situation with reduced parental support. Complaint narratives were rare, however, which could indicate that this might be a moral norm-breaking subject in a public chat blog. Hence, the identity position of being the loving and caring sister was most prominent in this context—perhaps at the price of not being able to fully share how the sibling’s cancer adversely affected their lives. At the same time, this self-positioning was grounded in these blogging sisters’ affectionate relationships with their ill siblings. Since young people in complicated or negative sibling relationships might hesitate to use this social arena for communication, there could be a self-selection with regard to *who* might construct a blog of this kind.

All three sisters, however, described their lonely position in coping with the illness situation, and how they were profoundly affected by the trauma of having a sister or brother suffer from cancer. They all described a “crisis of meaning” (Neimeyer & Sands, 2011), in which previous beliefs were no longer applicable, and they actively searched for new meanings through which the cancer event could be incorporated into their autobiography.

Three particularly vulnerable occasions—all described as turning points—were identified in this regard: (1) the sister or brother’s diagnosis with cancer; (2) the termination of treatment; and (3) the sister or brother’s death. What is noteworthy, however, is that even in the case where the ill sibling recovered, the sister reported that she was experiencing a lasting fear of relapse, which intensified in connection with the sibling’s health check-ups. These periods of elevated distress are important for health care professionals to be aware of, in order to provide adequate and timely sibling support.

The overall results highlight the need for siblings to narrate experience in a supportive context, preferably together with other siblings on the Internet. This finding is largely consistent with previous studies of online support groups for cancer patients and their relatives (see e.g., Coulson & Greenwood, 2012; Elder & Burke, 2015), and shows how such a community can be a useful avenue for connecting with others, sharing experiences and seeking support during periods of both illness and mourning.

## Methodological considerations

The narrative research approach chosen enabled an analysis of three sisters’ natural telling of their experiences of a sister or brother’s cancer in self-created contexts on the Internet. From their interaction with an audience consisting of supported readers of their blogs, it was possible to investigate their main concerns in their sibling position, their coping strategies, and their support needs—all of which provides valuable knowledge for improving professional practice. This can be compared to traditional research interviews, which are usually structured—and thus more easily steered—by the researcher’s own interests and agenda. Since siblings of a sister/brother with cancer have been described as an invisible group, whose suffering is commonly silenced by circumstances characteristic of the illness situation, a narrative methodology has been particularly useful for bringing voice to their experiences (Ribbens McCarthy, 2007; Riessman, 2008). The material is limited to three in-depth cases. This means that the results are based on unique experiences that simultaneously illustrate some general aspects of the phenomenon of being subjected to a sibling’s cancer in youth. The results should not, however, be interpreted as representative of *all* siblings. In addition, there is a selection bias in that only young women are represented. Hence, we cannot tell from the material how brothers or younger siblings might experience a sister/brother’s cancer.

## Conclusion

In this article, siblings’ need to process their relationship with the ill sister/brother has been shown to be a central element of their coping with cancer and death. This means that in their encounters with siblings—face-to-face or online—professionals are recommended to offer a space for communication in which they adhere to siblings’ own narrative coping strategies and when needed assist in the construction of alternative narrative framings that do not marginalize or stigmatize, but bring a manageable meaning to their experiences (cf. Eilertsen, Lövgren, Wallin, & Kreicbergs, 2017). This article shows that such a narrative context and such support can encourage siblings to break the silence and thus regain voice and agency, which would empower them in a situation that is otherwise largely characterized by their vulnerability.

## Note

1. Netnography is a collective term for the different ethnographic research methods used to analyze the behaviour of individuals on the Internet.
